# Cryoablation as local therapy in patients with locally recurrent thyroid cancer: Two case reports

**DOI:** 10.1016/j.radcr.2025.04.065

**Published:** 2025-05-08

**Authors:** Sorina R. Simon, Tim Lubbers, Sanne Engelen, Sanne W. de Boer, Christiaan van der Leij

**Affiliations:** a Department of Radiology and Nuclear Medicine, Maastricht University Medical Center, Maastricht 6229 HX, The Netherlands; b GROW—School for Oncology and Reproduction, Maastricht University, Maastricht, The Netherlands; c Department of Surgery, Maastricht University Medical Center, Maastricht, The Netherlands

**Keywords:** Thyroid cancer, Thyroid carcinoma, Cryoablation, Interventional oncology

## Abstract

The local surgical treatment of thyroid cancer recurrence poses significant challenges, particularly when addressing areas affected by extensive scar tissue. Cryoablation of local recurrences might be a promising minimally invasive alternative, especially for patients who are not ideal surgical candidates or prefer nonsurgical interventions. This report discusses 2 cases of recurrent thyroid cancer successfully managed with curative intent cryoablation. Follow-up ultrasound revealed complete involution in the first patient, and 73% volume reduction of the lesion in the second patient. No delayed complications were observed. It underlines the potential of cryoablation as a feasible treatment option for local recurrence of thyroid cancer and advocates for future research to better define its role within thyroid cancer management protocols.

## Introduction

Well-differentiated thyroid cancer is commonly treated with surgical resection and radioactive iodine therapy. Local recurrence remains a challenge, often requiring further surgical interventions. Extensive scar tissue from prior surgeries can complicate re-resection, especially in case of adhesions to critical structures like the trachea, oesophagus, recurrent nerves, or vessels [1]. Cryoablation could be a minimally invasive alternative to redo surgery in complex cases or patients avoiding additional surgery. Cryoablation utilizes freezing to induce ice crystal formation and subsequent cell death [2]. Previous research suggests that cryoablation is safe in the head and neck region when protective measures are taken [3]. The literature on cryoablation for locally recurrent thyroid cancer is limited, with only 1 study published to date [1]. Regarding its safety, the authors reported a major adverse event rate of 20%, including voice changes and Horner syndrome, though no permanent complications were observed during follow up [1]. Additionally, the study demonstrated a 100% response rate, with no local recurrences noted during follow-up [1].

This case report highlights the safe and successful use of curative intent cryoablation as a treatment option for locally recurrent thyroid cancer in 2 patients.

### Case 1

**Fig. 1:**
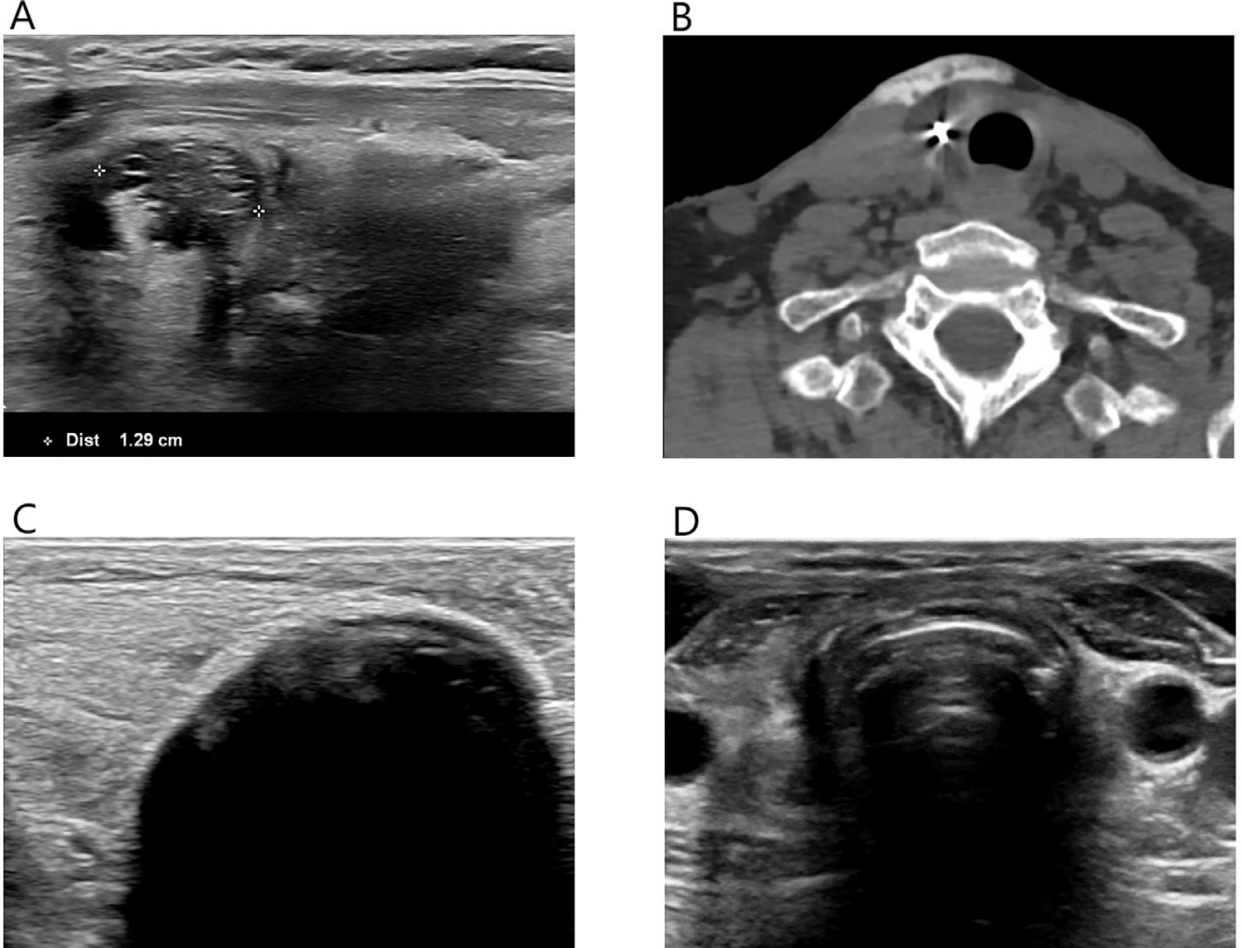
Treatment of recurrent Hurthle cell carcinoma. (A) Preprocedural ultrasound showing the local recurrence in the right thyroid bed. (B) Attempts at hydrodissection between the lesion and the trachea were unsuccessful due to adhesions, yet hydrodissection was successfully performed to protect the vessels and skin. This CT image also shows the cryoablation probe. (C) During cryoablation, the ice ball was expanded to achieve a 2 mm margin beyond the lesion. (D) Follow-up ultrasound after 3 months showed complete involution of the residual lesion.

A 75-year-old woman with a history of Hurthle cell carcinoma underwent total thyroidectomy in 2020, followed by radioactive iodine therapy for a pT3N0M0 tumor. Subsequent imaging revealed lymph node recurrence, leading to additional radioactive iodine therapy. In 2023, ultrasound detected recurrent Hurthle cell carcinoma in the right thyroid bed, confirmed by fine-needle aspiration. The patient underwent surgical resection in May 2023. In November 2023, ultrasound raised concerns for another recurrence in the right thyroid bed measuring 13 × 12 × 10 mm (Fig. 1A), confirmed by fine-needle aspiration (Bethesda VI). Treatment options were discussed, including repeated surgery or cryoablation. Curative intent cryoablation was selected as the preferred treatment due to the elevated risks associated with a complex re-resection. Cryoablation was performed under light sedation and local anesthesia with lidocaine to monitor recurrent laryngeal nerve function and prevent vocal cord paralysis. Hydrodissection failed to separate the lesion from the trachea due to adhesions, yet it was successfully performed to protect the vessels and skin (Fig. 1B). External heat packs were applied for skin warming. The cryoablation procedure was performed as described in the protocol outlined by Sag et al. [1]. A single cryoablation probe (14G IcePearl™ 2.1 CX, Boston Scientific, Marlborough, MA, USA) was placed under ultrasound guidance. Freezing commenced at 30% power and progressively increased with steps of 10% to 50% until the ice ball expanded to achieve a 2 mm margin beyond the lesion (Fig. 1C). The freezing cycle was performed for 10 minutes, with concurrent hydrodissection between the ice ball and skin. No voice changes occurred during the freezing phase, yet mild hoarseness developed during the thawing phase, which improved within minutes. Follow-up ultrasound after 1 month revealed a smaller hyperechoic residual lesion without evidence of local recurrence. By 3 months, the residual lesion had involuted completely on ultrasound, and the hoarseness had fully disappeared (Fig. 1D).

### Case 2

**Fig. 2:**
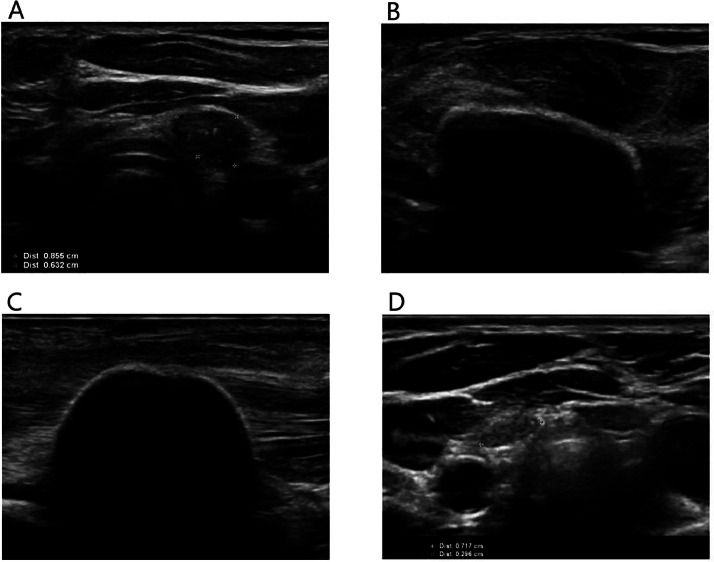
Treatment of recurrent papillary thyroid cancer. (A) Preprocedural ultrasound showing the local recurrence with coarse calcifications in the left thyroid bed. Attempts at hydrodissection to separate the lesion from the trachea were unsuccessful due to adhesions, but hydrodissection was successfully performed to protect the carotid artery and jugular vein. (B, C) The ice ball was monitored to ensure it extended at least 2 mm beyond the lesion margins. (D) Follow-up ultrasound at 7 months revealed a 73% volume reduction of the left thyroid bed lesion.

A 38-year-old female with a history of papillary thyroid cancer underwent total thyroidectomy with lymph node dissection in 2022. Pathology confirmed multifocal papillary thyroid cancer (pT2N1bMx). However, she developed hoarseness caused by left vocal cord paresis resulting from recurrent laryngeal nerve injury. Postoperative ultrasound identified residual thyroid tissue in the right thyroid bed, confirmed by I-123 SPECT/CT. Additional surgery was performed resulting in bilateral vocal cord paresis. Pathology of the excised tissue showed no evidence of residual cancer, and the patient underwent radioactive iodine ablation. Follow-up SPECT/CT demonstrated persistent iodine-avid tissue in the right thyroid bed. In 2023, ultrasound detected a new 9 × 6 × 10 mm nodule in the left thyroid bed (Fig. 2A), with cytology confirming recurrent papillary thyroid cancer. Curative intent cryoablation was selected as the preferred treatment due to pre-existing bilateral vocal cord paresis and the patient’s preference to avoid additional surgery. The procedure was performed under light sedation and local anesthesia. Hydrodissection failed to separate the lesion from the trachea due to adhesions but was successfully performed to protect the carotid artery and jugular vein (Fig. 2B). Cryoablation was, again, performed as described in the protocol outlined by Sag et al. [1]. A single cryoablation probe (14G IcePearl™ 2.1 CX, Boston Scientific, Marlborough, MA, USA) was placed into the lesion, and freezing commenced at 30% power, gradually increasing with steps of 10% to 50%. The ice ball was monitored to ensure it extended at least 2 mm beyond the lesion margins (Fig. 2C). Cryoablation was maintained at 50% power for 10 minutes. The patient tolerated the procedure well, with preserved vocal function during and after the procedure. Follow-up ultrasound at 1 month showed a size reduction to 8 × 4 × 9 mm, which further decreased to 8 × 3 × 7 mm at 3 months. Postablation ultrasound at 7 months revealed a 73% volume reduction of the left thyroid bed lesion, measuring 7 × 3 × 7 mm (Fig. 2D). No immediate or delayed complications were observed.

## Discussion

The treatment of local recurrence of thyroid cancer can be complex, particularly when extensive scar tissue or adhesions to critical structures such as the trachea, nerves, or vessels hinder re-resection. Repeated surgery has been associated with complication rates of up to 11% [4]. While thermal ablation techniques like RFA have been used in the head and neck region for benign thyroid nodules, thermal ablation carries risks of thermal injury to surrounding vessels, nerves, and airways [1]. Cryoablation offers a promising minimally invasive alternative, particularly for patients with surgical risks or preferences against additional surgery. Cryoablation provides precise control over the ablation zone through the visualization of ice formation. Its unique ability to preserve collagen structures distinguishes it from other ablation techniques [2]. Additionally, cryoablation can reduce nerve activity and pain signals, improving patient comfort and procedural tolerance [2]. Its safety profile is particularly advantageous near critical structures such as vessels, nerves, and airways, potentially minimizing the risk of permanent injury [5].

This report indicates that cryoablation might be a safe and feasible treatment option for locally recurrent thyroid cancer. In this case report, the patients were treated following the protocol outlined by Sag et al. [1], which included ensuring a minimum of 2 mm ice overcoverage for the lesion, applying external heat packs for skin warming, and performing only 1 freezing cycle. Protective techniques, such as hydrodissection, were attempted to safeguard surrounding structures. Although hydrodissection was unsuccessful in separating the lesion from the trachea in both patients, it did not lead to tracheal damage during follow-up. This may be attributed to the ability of cryoablation to preserve collagen structures, as collagen fibers are more resistant to freezing damage compared to other cellular components. This characteristic suggests that cryoablation may be a safer approach for treating lesions near cartilage or other collagen-rich tissues, including the trachea [6]. Similarly, Sag et al. reported no long-term tracheal complications after failed hydrodissection in all cases which underlines this theory. Regarding other adverse events, 1 patient experienced temporary mild hoarseness which resolved over time. No complications involving vascular, tracheal, dermal, or infectious issues were observed in either patient, in line with the report by Sag et al. [1]. During follow-up, 1 patient demonstrated complete involution of the lesion, while the second showed a 73% reduction in lesion volume. No local tumor progression was observed, consistent with the findings of Sag et al., who reported complete involution in 60% of cases and partial involution in 40% [1].

Future studies should further explore and define the role of cryoablation in the treatment of locally recurrent thyroid cancer, focusing on its safety, efficacy, and long-term outcomes.

## Conclusion

Cryoablation is a promising minimally invasive approach for treating locally recurrent thyroid cancer, particularly in patients where re-resection is difficult or contra-indicated due to extensive scar tissue. These cases underline the importance of individualized treatment planning and the potential role of advanced ablative techniques in thyroid oncology.

## Ethical approval

All procedures performed in studies involving human participants were in accordance with the ethical standards of the institutional and/or national research committee and with the 1964 Helsinki declaration and its later amendments or comparable ethical standards.

## Patient consent

Written informed consent was obtained from the patients for publication of this case report and any accompanying images.
